# Hepatitis C Virus Mediated Changes in miRNA-449a Modulates Inflammatory Biomarker YKL40 through Components of the NOTCH Signaling Pathway

**DOI:** 10.1371/journal.pone.0050826

**Published:** 2012-11-30

**Authors:** Nayan J. Sarma, Venkataswarup Tiriveedhi, Vijay Subramanian, Surendra Shenoy, Jeffrey S. Crippin, William C. Chapman, Thalachallour Mohanakumar

**Affiliations:** 1 Department of Surgery, Washington University School of Medicine, Saint Louis, Missouri, United States of America; 2 Department of Pathology & Immunology, Washington University School of Medicine, Saint Louis, Missouri, United States of America; 3 Department of Medicine, Washington University School of Medicine, Saint Louis, Missouri, United States of America; National Institutes of Health, United States of America

## Abstract

Liver disease due to hepatitis C virus (HCV) infection is an important health problem worldwide. HCV induced changes in microRNAs (miRNA) are shown to mediate inflammation leading to liver fibrosis. Gene expression analyses identified dysregulation of miRNA-449a in HCV patients but not in alcoholic and non-alcoholic liver diseases. By sequence analysis of the promoter for *YKL40,* an inflammatory marker upregulated in patients with chronic liver diseases with fibrosis, adjacent binding sites for nuclear factor of Kappa B/P65 and CCAAT/enhancer-binding protein alpha (CEBPα) were identified. P65 interacted with CEBPα to co-operatively activate *YKL40* expression through sequence specific DNA binding. *In vitro* analysis demonstrated that tumor necrosis factor alpha (TNFα) mediated *YKL40* expression is regulated by miRNA-449a and its target *NOTCH1* in human hepatocytes.NOTCH1 facilitated nuclear localization of P65 in response to TNFα. Further, HCV patients demonstrated upregulation of *NOTCH1* along with downregulation of miRNA-449a. Taken together it is demonstrated that miRNA-449a plays an important role in modulating expression of *YKL40* through targeting the components of the NOTCH signaling pathway following HCV infection. Therefore, defining transcriptional regulatory mechanisms which control inflammatory responses and fibrosis will be important towards developing strategies to prevent hepatic fibrosis especially following HCV recurrence in liver transplant recipients.

## Introduction

Liver diseases resulting from hepatitis C virus (HCV) infection is a major health issue worldwide as well as the United States [1], [2]. It is estimated that about 4 million people are infected with HCV in the United States and about 300 million worldwide [1]. The natural history of HCV infection in the liver is characterized by slow progression to fibrosis and cirrhosis, end-stage liver diseases, and high risk of developing hepatocellular carcinoma (HCC) [3].

YKL40 (CHI3L1) is a member of the “mammalian chitinase–like proteins,” secreted by activated macrophages and neutrophils during inflammation in various tissues including liver, smooth muscle and cancer cells [4]. YKL40 is elevated in patients with chronic liver diseases that are characterized by inflammation and increased extra-cellular remodeling [5], [6]. Although increased levels of YKL40 have been shown to be induced by tumor necrosis factor alpha (TNFα), the molecular mechanisms are not clearly identified [7]. TNFα, an inflammatory cytokine regulates gene expression in the nuclear factor of Kappa B (NFKB) signaling pathway [8]. Components of the mammalian NFKB family of transcription factors includes NFKB1 (P105/P50), NFKB2 (P100/P52), RelA (P65), RelB and c-Rel [9]. The NFKB component P65 is a multimeric DNA binding transcription factor involved in inflammatory and immune disorders especially autoimmune diseases and cancer [10]. NOTCH1 is one of the upstream regulator of NFKB complex and downregulation of NOTCH1 impairs its function [11], [12]. It has been shown that NOTCH1 and TNFα regulate nuclear retention of NFKB [13], [14]. CCAAT/enhancer-binding protein alpha (CEBPα) is a homodimeric DNA binding bZIP transcription factor that controls cell proliferation and differentiation [15]. CEBPα is differentially regulated in cases of HCC and targets expression of a wide range of genes and microRNAs (miRNA) involved in liver diseases [16], [17].

miRNAs have been shown to play an important role in immune evasion, regulation of cell cycle and in cancer progression [18], [19], [20]. HCV infection results in modulation of miRNA particularly those that control viral particle entry and propagation, thus playing an important role in host immune evasion [21]. In this study we defined the molecular mechanisms of *YKL40* expression that involves HCV induced miRNA modulation and regulation by novel pathways including NOTCH1, NFKB and CEBPα.

**Table 1:** Patient Demographics.

Demographic		HCV	AH	NASH	Control
Number (n)		10	10	10	10
Age (mean, SD)		57±4	50±7	54±5	34±13
Gender M:F		6∶4	5∶5	7∶3	5∶5
Recipient Race (n)	Caucasian	5	4	5	5
African American		5	5	5	5
	Others		1		
Bilirubin (mg/dL)		1.9±0.4	1.5±0.3	1.8±0.6	0.9±0.8
AST (IU/ml)		90±34	110±40	74±30	25±10
ALT (IU/ml)		55±29	140±60	63±38	23±12
HCV Viral load (X10^6^/mL)		1.4±0.3	n/a	n/a	n/a
HCV genotype	1	4	n/a	n/a	n/a
	%	21.5%	n/a	n/a	n/a
	1a	4	n/a	n/a	n/a
	%	30.8%	n/a	n/a	n/a
	1b	2	n/a	n/a	n/a
	%	15.4%	n/a	n/a	n/a

SD: Standard Deviation, HCV: Hepatitis C Virus, AH: Alcoholic Hepatitis, NASH: Non-Alcoholic Steatohepatitis, M: Male, F: Female, AST: Aspartate Amino Transferase, ALT: Alanine Amino Transferase.

## Materials and Methods

### Patients

Liver biopsies were obtained from 10 chronic HCV patients, 10 alcoholic hepatitis patients, 10 non-alcoholic steatohepatitis patients and 10 normal donor livers (control) at the time of transplantation at Washington University Medical Center/Barnes-Jewish Hospital (Table 1). Patients with hepatitis B virus and/or HIV were excluded from the study. All of the human studies were approved by the human research protection committee at Washington University (protocol 201104075) and patients were enrolled after written informed consent was obtained.

**Figure 1:**
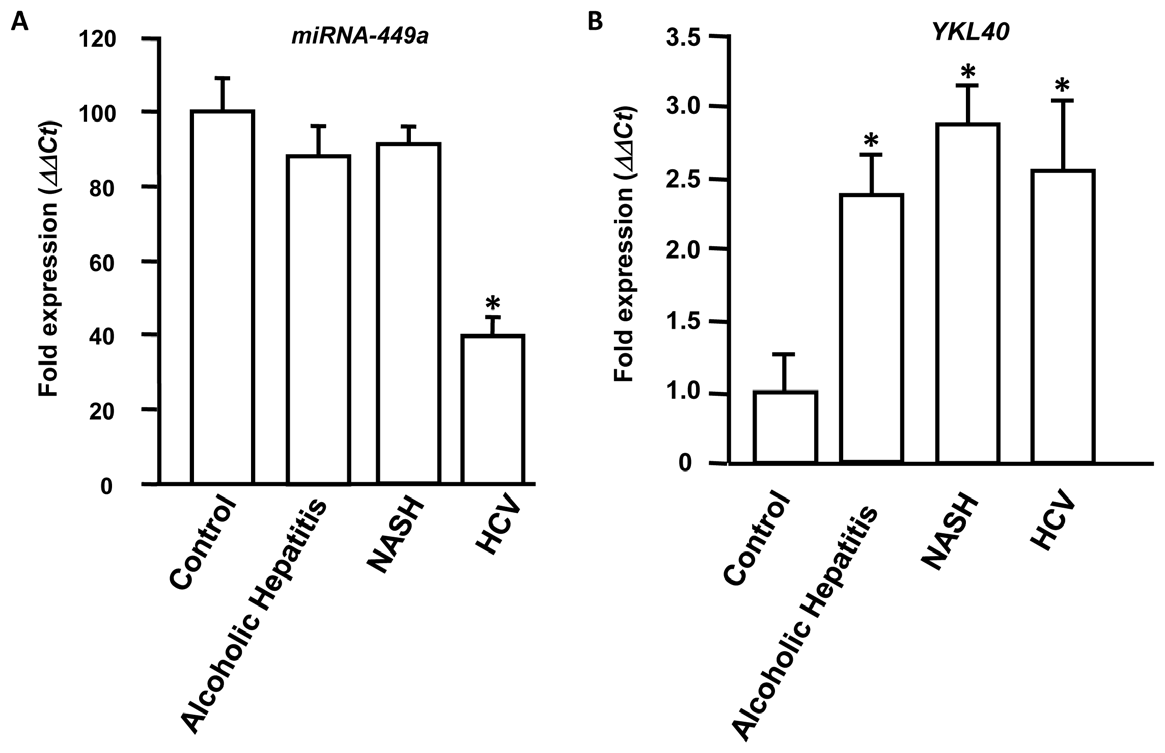
miRNA-449a is downregulated in HCV patients and *YKL40* is upregulated in patients with hepatic fibrosis. Total RNA was isolated from liver biopsies obtained from 10 chronic HCV patients, 10 alcoholic hepatitis patients, 10 non-alcoholic steatohepatitis (NASH) patients and 10 normal donor livers (control). Expression of miRNA-449a (1A) and *YKL40* (1B) were determined by Q-PCR. The *ΔΔCt* value was calculated by normalizing the threshold (CT) values with *GAPDH* expression and miRNA-449a (1A) or *YKL40* (1B) expression respectively in controls. The ‘*’ represents p value<0.01 obtained by a two-tailed t test. Error bars represent Standard Deviations (SD) calculated from three independent experiments.

### Plasmids and Constructs

For *YKL40* luciferase constructs, the promoter regions were amplified from human genomic DNA (Zyagen, CA) by PCR using iProof High-Fidelity DNA Polymerase (Biorad, CA). PCR products were subcloned into pGL4.11 vector (Promega, WI) upstream of a luciferase gene using the NheI/EcoRV restriction sites. P65 and CEBPα were amplified from a human cDNA library (Stratagene, CA) and subcloned into pcDNA using the HindIII/Not1 and HindIII/BamH1 restriction sites, respectively. Hsa-miRNA-449a (SC400399) and control constructs were purchased from Origene, MD. *NOTCH1* (sc-36095), P65 (sc-29410) and control siRNA (sc-37007) were purchased from Santacruz Biotechnology, CA. Computational analysis of the promoter bound transcription factors was done using the Transcription Element Search System http://www.cbil.upenn.edu/cgi-bin/tess/tess. miRNA target analysis was done using http://www.targetscan.org.

**Figure 2:**
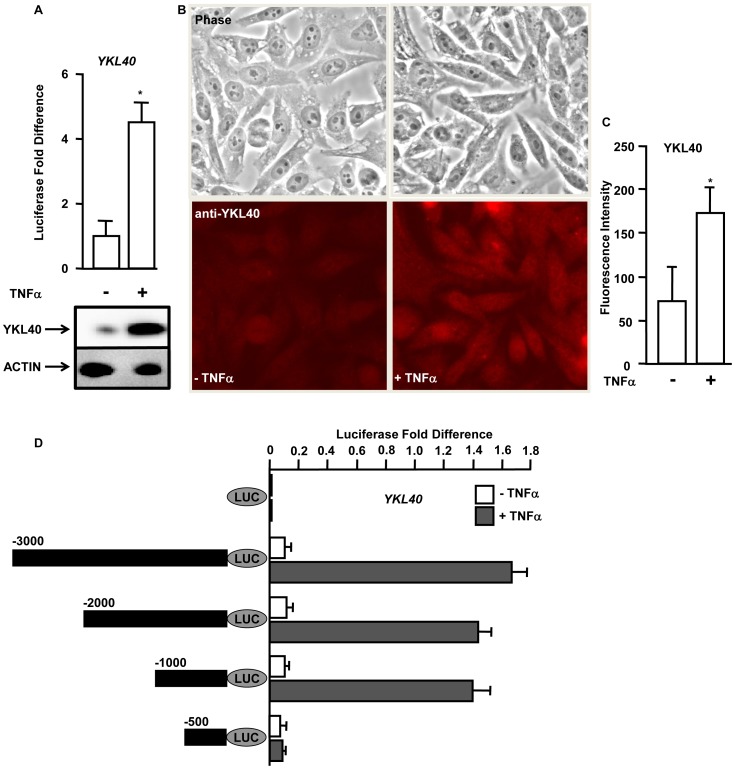
TNFα regulates the expression of *YKL40* at the transcriptional level. A. (*Upper panel*) Hepatocytes were transfected with an −3000 bp *YKL40*-promoter driven reporter construct with (+) or without (−) TNFα. Firefly luciferase activity was measured 48 hours after transfection and normalized to a Renilla luciferase internal control. The numbers represent fold-change over control (average of three independent experiments); error bars represent SD. The ‘*’ represents p value<0.05 obtained by a two-tailed t test. (*Lower panel*): hepatocytes from 2A were immunoblotted with anti-YKL40 with (+) or without (−) TNFα. ACTIN was used as the loading control. B. HEPG2 cells were immunostained with anti-YKL40 antibody without (−) or with (+) TNFα. C. Quantification of the YKL40 immunostaining signal in HEPG2 cells (1B). The numbers represent the average fluorescence intensity of YKL40 (n = 100). D. Essential regions in the *YKL40* promoter required for TNFα mediated expression. Hepatocytes were transfected with luciferase reporters driven by deletion constructs of *YKL40* promoter (−3000 bp, −2000 bp, −1000 bp, −500 bp, filled black bars on left) construct with (+) or without (−) TNFα. Firefly luciferase activity was measured 48 hours after transfection and normalized to a Renilla luciferase internal control. The luciferase activity was normalized to the control empty luciferase vector and the numbers represent fold-change over control (average of three independent experiments); error bars represent SD calculated from three independent experiments.

### miRNA and mRNA Expression Analysis

Total RNA was isolated from the liver biopsies or hepatocytes using the RNAaqueous kit (Ambion, NY). Expression level of miRNA-449a was determined using the TaqMan® MicroRNA assays and TaqMan® Universal Master Mix II (Life technologies, NY) using predesigned primers. Quantitative PCR (qPCR) to analyze *YKL40* and *NOTCH1* was performed using a BioRad Real-Time PCR System with cycling conditions of 95°C for 10 min followed by 95°C for 15 sec and 60°C for 60 sec for a total of 40 cycles. Each TaqMan assay was run in triplicate. The *ΔΔCt* value was calculated by normalizing the threshold (CT) values with *GAPDH* expression and respective gene expression in controls.

**Figure 3:**
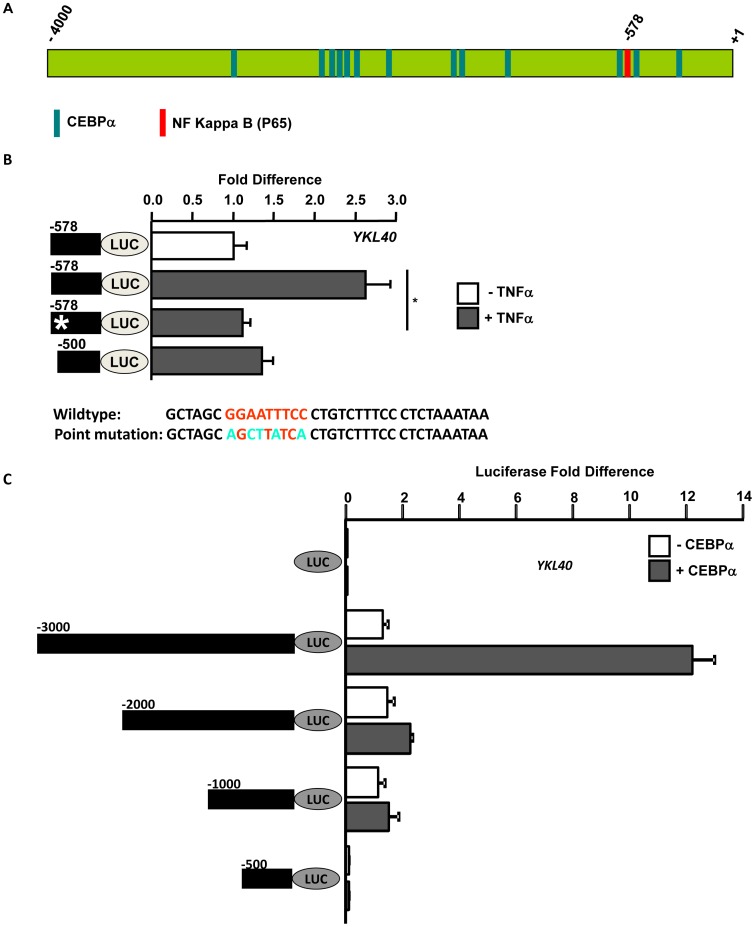
Transcription factors P65 and CEBPα regulate expression of *YKL40.* A. Computational prediction of transcription factors binding to the *YKL40* promoter. Computational analysis of −4000 bp upstream of the open reading frame using the Transcription Element Search System (TESS). The bars represent the predicted consensus binding sites in the DNA for transcription factors P65 and CEBPα. B. Mutation of NFKB/P65 binding site inhibits TNFα mediated *YKL40* induction. Hepatocytes were transfected with luciferase reporters driven by deletion constructs of *YKL40* promoter −578 bp wildtype, −578 bp P65 binding site mutated (*) and −500 bp wildtype (P65 site deleted), filled black bars on left) construct with (+) or without (−) TNFα. Firefly luciferase activity was measured 48 hours after transfection and normalized to a Renilla luciferase internal control. The numbers represent fold-change over the −578 wildtype construct without TNFα treatment (average of three independent experiments); error bars represent SD. The P65 binding site mutation is shown in the lower panel. The ‘*’ represents p value <0.05 obtained by a two-tailed t test. C. CEBPα in an upstream transcription factor to activate *YKL40* expression. Hepatocytes were transfected with luciferase reporters driven by deletion constructs of *YKL40* promoter (−3000 bp, −2000 bp, −1000 bp, −500 bp, filled black bars on left) along with an empty vector or vector expressing CEBPα and treated with TNFα. Firefly luciferase activity was measured 48 hours after transfection and normalized to a Renilla luciferase internal control. The numbers represent fold-change over the control empty luciferase vector (average of three independent experiments); error bars represent SD.

### Primary Hepatocytes and HEPG2 Cell Line Transfection

Primary human hepatocytes were purchased from Life Technologies, New York and grown in 24 well plates in Williams Medium E supplemented with 5% FCS, 100 units/mL penicillin, 100 units/ml Amphotericin, 0.1% Albumin, 300 nM insulin, 2 mM L-glutamine and 0.1 nM Hydrocortisone. HEPG2 cells were grown in RPMI with 10% FBS, 1 mM sodium-pyruvate, 10 mM HEPES, 2 mM L-glutamine, and 100 units/mL penicillin/streptomycin. 20 ng/ml of TNFα (Sigma, St. Louis, MO) was added for 6 hours wherever indicated. The optimal concentration of TNFα was determined by dose-dependent analyses.

**Figure 4:**
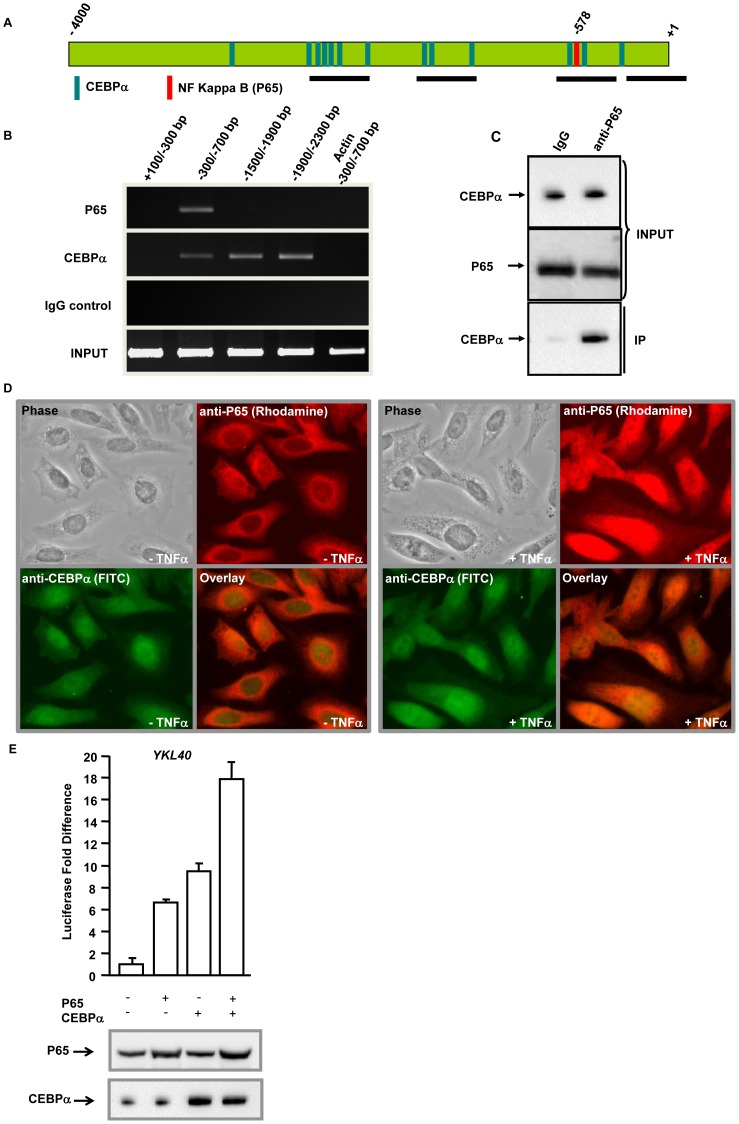
CEBPα interacts with NFKB/P65 to bind *YKL40* promoter and cooperates to activate transcription in hepatocytes. A. *YKL40* promoter showing binding sites for P65 and CEBPα. The black horizontal bars represent regions amplified by the PCR primers. B. Chromatin was immunoprecipitated with anti-P65 or anti-CEBPα or isotype control IgG from hepatocytes. Segments of the *YKL40* promoter (indicated in 4A) were amplified by PCR. The first three lanes show immunoprecipitated chromatin (IP) and the fourth lane show input chromatin (Input). *ACTIN* promoter amplification is shown as the negative control. C. Co-immunoprecipitation of P65 with CEBPα in hepatocytes. Whole cell lysates were subjected to immunoprecipitation with either rabbit IgG or anti-P65. CEBPα in the cell lysates (Input) and immunoprecipitated complexes (IP) was detected by immunoblotting with anti-CEBPα. P65 was detected by immunoblotting with anti-P65. D. HEPG2 cells were treated with (+) or without (−) TNFα and co-immunostained with anti-CEBPα and anti-P65. E. Hepatocytes were transfected with a luciferase construct driven by the −3000 bp *YKL40* promoter in addition to the control vector or vector expressing P65 or CEBPα or both. Firefly luciferase activity was measured 48 hours after transfection and normalized to a Renilla luciferase internal control. The numbers represent fold-change over the control empty vector (average of three independent experiments); error bars represent SD. Bottom panel, expression of P65 and CEBPα was verified by immunoblotting with anti-P65 and anti-CEBPα respectively.

For transfection of hepatocytes and HEPG2 cells (ATCC), 0.2×10^5^ cells were seeded into each well of a 24 well plate and grown for 24–48 hours. On the day of transfection, medium was changed and 500 ng of DNA was transfected using Lipofectamine™ LTX and Plus Reagent (Invitrogen, NY). For siRNA delivery 0.1×10^5^ cells were grown in each well of a 24 well plate for 24–48 hours in antibiotic free medium and 80 pico moles of siRNA were transfected using Lipofectamine™ RNAiMAX (Invitrogen). Cells were harvested 48 hours post transfection and efficiency was measured by qPCR, immunostaining and western blot.

**Figure 5:**
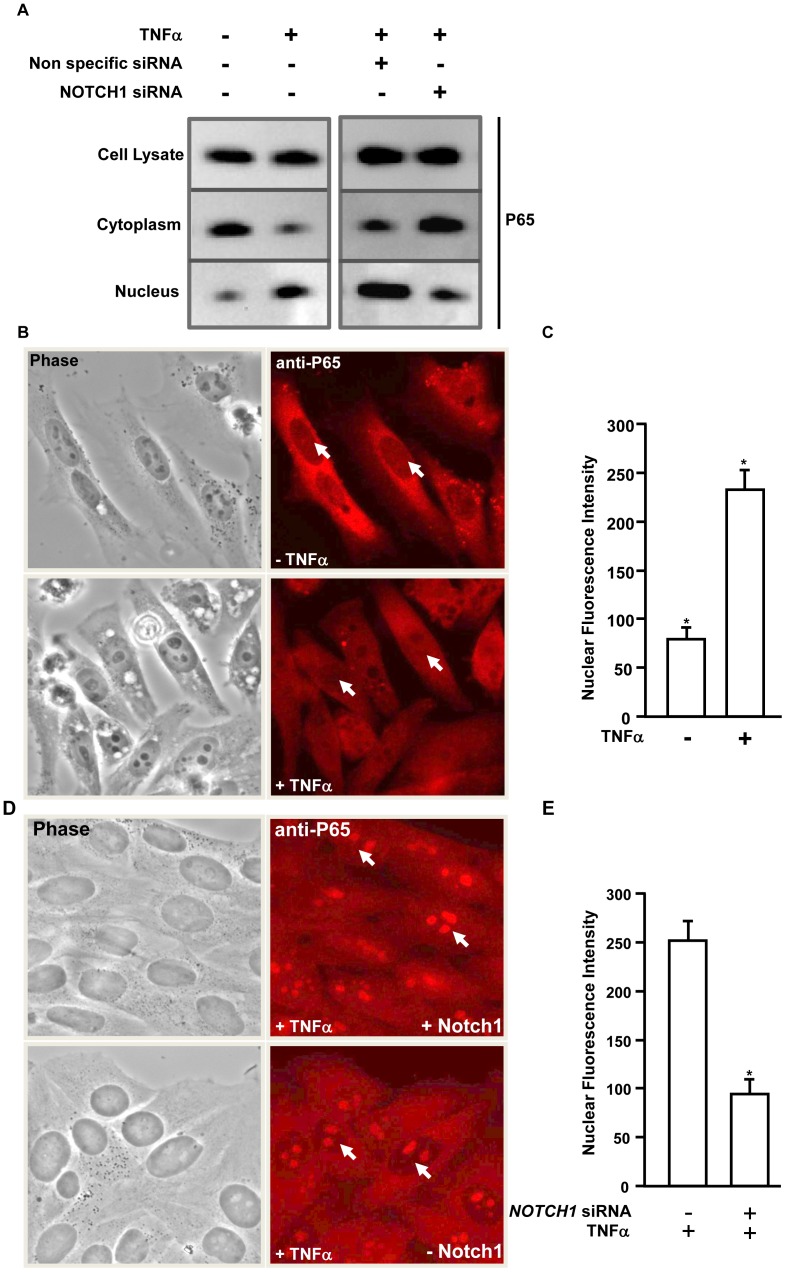
Notch1 regulates nuclear retention of NFKB/P65 in response to TNFα. A. (*Right panel*) Hepatocytes were treated with (+) or without (−) TNFα and cytoplasmic and nuclear fractions were extracted. (*Left panel*) hepatocytes were transfected with either non-specific siRNA or siRNA specific for NOTCH1 and treated with TNFα and cytoplasmic and nuclear fractions were extracted. Whole cell lysates, cytoplasmic and nuclear extracts were subjected to immunoblotting with anti-P65. B. HEPG2 cells were treated with (+) or without (−) TNFα and immunostained with anti-P65. The arrows indicate nuclear localization of P65. C. Quantification of the P65 immunostaining signal in HEPG2 cells (5B). The numbers represent the average fluorescence intensity of P65 (n = 100). D. HEPG2 cells were transfected with either non-specific siRNA or siRNA specific for NOTCH1 and treated with TNFα. The arrows indicate nuclear localization of P65. E. Quantification of the P65 immunostaining signal in HEPG2 cells (5D). The numbers represent the average fluorescence intensity of P65 (n = 100). The ‘*’ represents p value<0.05 obtained by a two-tailed t test.

### Western Blot and Immunofluorescence Microscopy

For localization of YKL40, NOTCH1 and P65, 50,000 HEPG2 cells were grown on coverslips in 24 well plates. Immunostaining was done as described before [22]. Briefly, cells were fixed with 4% paraformaldehyde and permeabilized with 0.1% Triton X-100. 2% normal goat serum in DPBS with 1% BSA, 0.1% Tween 20 was used for blocking and washing. Primary antibodies for western blot and Immunofluorescence used were goat anti-YKL40 (sc-31722), rabbit anti-NOTCH1 (sc-9170), mouse anti-CEBPα (sc-166258) and rabbit anti-P65 (sc-109). The secondary antibodies used were FITC-conjugated anti-mouse IgG (sc-2010), Rhodamine-conjugated anti-goat IgG (sc-3945) and Rhodamine-conjugated anti-rabbit IgG (sc-2492). The images were captured using an Eclipse 80i fluorescent microscope (Nikon, NY) and processed using Metamorph version 6.3r2 software (Molecular Devices, CA). Extraction of the nuclear and cytoplasmic fractions from the hepatocytes (1×10^6^ cells) was done using NE-PER® Nuclear and Cytoplasmic Extraction Kit (Thermo Scientific, IL).

**Figure 6:**
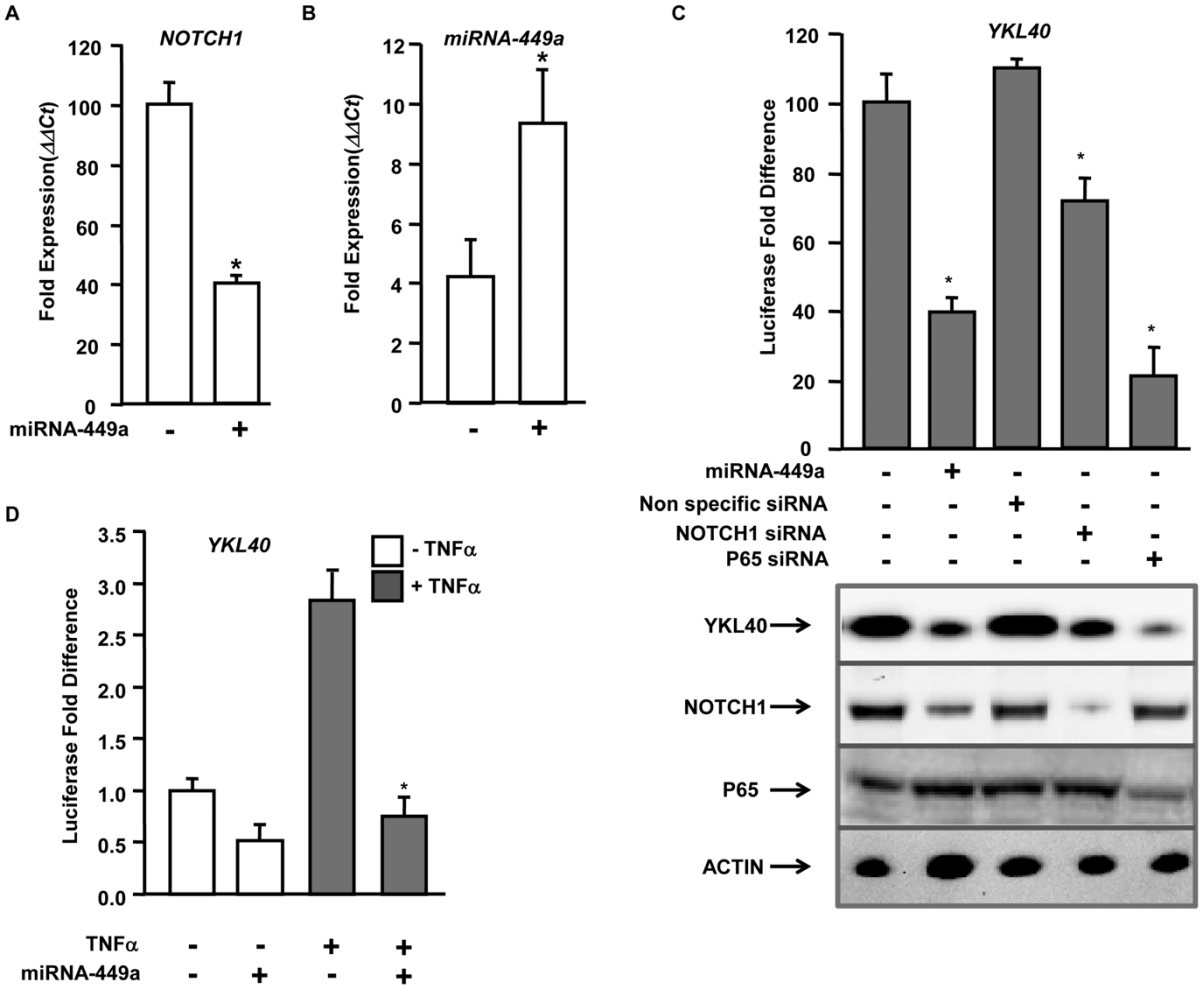
microRNA 449a regulates *YKL40* expression by targeting *NOTCH1* for silencing. A & B. Hepatocytes were transfected with an empty vector (−) or vector expressing miRNA-449a (+) and expression of *NOTCH1* (6A) and miRNA-449a (6B) were determined by Q-PCR. The *ΔΔCT* value was calculated by normalizing the threshold (CT) values with *GAPDH* and expression of *NOTCH1* and miRNA-449a respectively in controls. The ‘*’ represents p value<0.05 obtained by a two-tailed t test. C. (*Upper panel*) hepatocytes were transfected with a luciferase construct driven by the *YKL40* promoter in addition to the control vector or vector expressing miRNA-449a or non-specific siRNA or siRNA specific for *NOTCH1* or siRNA specific for *P65* in the presence of TNFα. Firefly luciferase activity was measured 48 hours after transfection and normalized to a Renilla luciferase internal control. The numbers represent fold-change over the control vector (average of three independent experiments); error bars represent SD. (*Lower panel*) Downregulation of YKL40, NOTCH1 and P65 is verified by immunoblotting with anti-YKL40 or anti-NOTCH1 or anti-P65 respectively. ACTIN is shown as the loading control. D. Hepatocytes were transfected with a luciferase construct driven by the *YKL40* promoter in addition to the control vector or vector expressing miRNA-449a construct with (+) or without (−) TNFα. Firefly luciferase activity was measured 48 hours after transfection and normalized to a Renilla luciferase internal control. The numbers represent fold-change over the control vector without TNFα (average of three independent experiments); error bars represent SD. The ‘*’ represents p value<0.01 obtained by a two-tailed t test.

### Luciferase Assay

Human primary hepatocytes (1×10^5^) were transfected in 24 well plates as mentioned earlier with 1 µg pGL4.11 luciferase reporter vector or pGL4.11 driven by the *YKL40* promoter or deletion constructs. For miRNA regulation studies the reporter construct was transfected in combination with 1 µg of either control vector or vector expressing miRNA-449 precursor. For *NOTCH1* regulation studies the reporter construct was transfected in combination with 80 picomoles of either non specific siRNA or siRNA specific for *NOTCH1*. For transcription factor studies the reporter constructs were transfected in combination with 2 µg of empty pcDNA3 vector, or pcDNA3 expressing P65 or CEBPα or both. 20 ng/ml of TNFα was added to the medium 6 hours before harvesting. To control for efficiency of transfection, 0.1 µg of pRL-TK (Promega, Madison, WI), which expresses Renilla luciferase was included. Luciferase activity was measured 48 h after electroporation using the Dual Luciferase Reporter Assay System (Promega, Madison, WI) and the results were normalized to Renilla luciferase.

**Figure 7:**
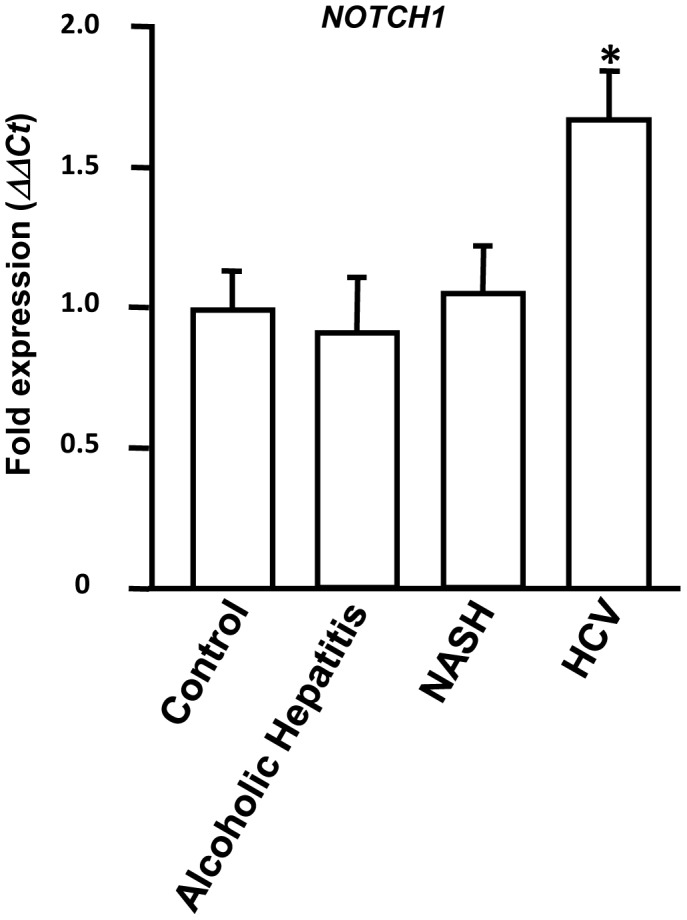
*NOTCH1* expression is upregulated in HCV patients. Total RNA was isolated from liver biopsies obtained from 10 chronic HCV patients, 10 alcoholic hepatitis patients, 10 NASH patients and 10 normal donor livers (control). Expression of *NOTCH1* was determined by Q-PCR. The *ΔΔCT* value was calculated by normalizing the threshold (CT) values with *GAPDH* expression and expression of *NOTCH1* in controls. The ‘*’ represents p value<0.01 obtained by a two-tailed t test. Error bars represent SD.

### Co-immunoprecipitation

Immunoprecipitation of P65 with CEBPα or the reverse immunoprecipiation in TNFα treated hepatocytes (1×10^6^) were carried out as described by Sarma et al [22]. Briefly, cells were washed with DPBS, and lysed with 0.5 ml of lysis buffer (10 mM Tris-HCl, pH 7.5, 0.4 M NaCl, 1% Nonidet P-40, 0.4% Triton X-100, 0.2% sodium deoxycholate, 1 mM EDTA, protease inhibitors (PI), 1 mM PMSF). Diluted with 0.5 ml buffer containing 10 mM Tris-HCl, pH 7.5, 1 mM EDTA, PI, 1 mM PMSF and centrifuged at 17,000×g for 30 min. 1 µg normal mouse/rabbit IgG or mouse anti-CEBPα or rabbit anti-P65 was used to immunoprecipitate the complexes from the supernatant. After overnight incubation with the antibodies 30 µl of Protein G beads were added to lysates and incubated for another 1 hour. Beads were washed with 700 µl of wash buffer (10 mM Tris-HCl, pH 7.5, 0.2 M NaCl, 0.5% Nonidet P-40, 0.2% Triton X-100, 0.1% sodium deoxycholate, 1 mM EDTA, PI, 1 mM PMSF) 3 min each for 5 times and once with cold DPBS by centrifugation at 1,800×g for 3 min at 4°C. Immunocomplexes were eluted by boiling with 30 µl of 2X SDS buffer (0.1 M Tris-HCl, pH 6.8, 3.5% SDS, 10% glycerol, 2 mM DTT, 0.004% bromphenol blue) for 10 min and subjected to SDS-PAGE (4–20% gel). P65 or CEBPα were detected with a rabbit anti-P65 or a mouse anti-CEBPα respectively.

**Figure 8:**
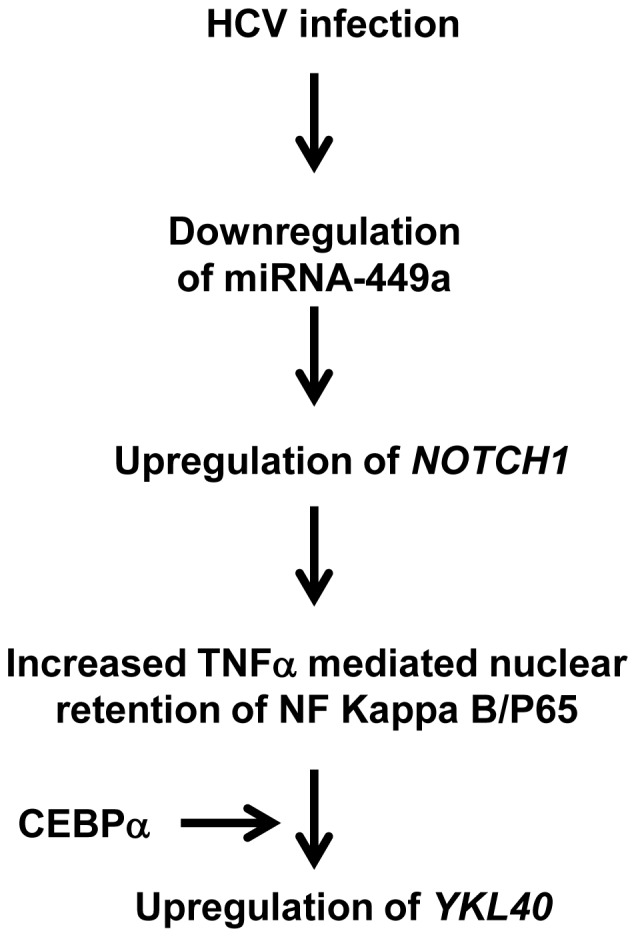
Schematic representation of HCV mediated role of miRNA-449a in *YKL40* expression. HCV infection results in downregulation of miRNA-449a that leads to upregulation of *NOTCH1* eventually results in nuclear stabilization of P65. P65 upregulates *YKL40* expression in co-operation with CEBPα in response to TNFα.

### Chromatin Immunoprecipitation

Chromatin Immunoprecipitation was carried out with ChIP-IT™ Express (Active Motif, Carlsbad, CA). Briefly, hepatocytes (1×10^6^) were crosslinked with 1% formaldehyde and quenched with 0.375 M glycine. Nuclei were isolated and sonicated in 350 µl of shearing buffer to prepare chromatin extracts. 1 µg of antibodies for control IgG, P65 or CEBPα were added to 60 µl of sheared chromatin along with Protein G Magnetic Beads. Antibody-lysate mix was washed and DNA was eluted according to the instructions. *YKL40* promoter regions were amplified by PCR.

## Results

### miRNA-449a is Downregulated in HCV Patients

A genomewide miRNA analysis in liver biopsies obtained from chronic HCV infected patients demonstrated a distinct expression profile when compared to the normal liver. Particularly, a significant downregulation of microRNA-449a was observed in the HCV infected livers.

To analyze the specific role of miRNA-449a following HCV infection, biopsies were obtained from 10 chronic HCV patients, 10 alcoholic hepatitis patients, 10 non-alcoholic Steatohepatitis (NASH) patients and 10 control normal donor livers at the time of liver transplant. Total RNA was isolated from the liver biopsies and expression level of miRNA-449a was determined by qPCR using specific primers and the results were normalized to *GAPDH* expression. Expression analysis demonstrated that miRNA-449a is downregulated more than two fold in livers obtained from HCV patients whereas no significant differences in the expression was observed in alcoholic hepatitis patients, NASH patients and normal livers (Figure 1A). This suggests that miRNA-449a is specifically downreguated in patients with liver diseases following HCV infection.

### *YKL40* is Upregulated in HCV Patients with Fibrosis

To determine the expression level of *YKL40* in liver diseases, biopsies were obtained from 10 chronic HCV patients, 10 alcoholic hepatitis patients, 10 NASH patients and 10 control normal donor livers at the time of liver transplant. Total RNA was isolated from the liver biopsies and expression level of *YKL40* was determined by qPCR using specific primers and the results were normalized to *GAPDH* expression. Expression analysis showed that *YKL40* to be upregulated in alcoholic hepatitis patients (2.4 fold), NASH patients (2.9 fold) and HCV patients (2.6 fold) compared to normals (Figure 1B). These results demonstrate that *YKL40* is elevated in patients with chronic liver diseases which are accompanied by inflammation.

### TNFα Regulates the Expression of *YKL40* at the Transcriptional Level

We and others have shown that *YKL40* expression is elevated in patients with chronic liver diseases accompanied by inflammation [5], [6]. *In vitro* studies have shown that inflammatory cytokines such as TNFα have the ability to induce expression of *YKL40* [7]. To study the molecular mechanisms of TNFα mediated regulation of *YKL40,* −3000 base pairs (bp) of the human *YKL40* promoter was cloned upstream of a luciferase reporter gene and the construct was introduced into human hepatocytes. The cells were treated either with or without 20 ng/ml TNFα for 6 hours and luciferase activity was measured. More than four fold increase in the *YKL40* promoter activity was observed in cells treated with TNFα compared to untreated (Figure 2A, upper panel). Further, western blot analysis showed increased expression of YKL40 in TNFα treated cells (Figure 2A, lower panel). This reporter gene analysis suggests that TNFα regulates the expression of *YKL40* through modulation of upstream transcriptional complexes that interact with the *YKL40* promoter.

To further demonstrate that TNFα induces expression of *YKL40*, human HEPG2 cells were cultured and treated with TNFα. Immunostaining of the HEPG2 cells with anti-YKL40 showed a more than 2 fold increase in expression of YKL40 in the cells treated with TNFα compared to untreated cells (Figures 2B and 2C).

### Reporter Analysis Identified Essential Regions in the *YKL40* Promoter Required for TNFα Regulated Expression of YKL40

Since TNFα regulated the expression of *YKL40* at the transcriptional level we hypothesized the possible interaction of TNFα with upstream regulatory complexes of the *YKL40* gene. To identify the essential regions for TNFα mediated expression deletion mutants of the *YKL40* promoter regions were cloned upstream of a luciferase reporter gene. Sequential deletion mutants of the *YKL40* promoter region (Figure 2D, black filled bars on the left) were introduced into hepatocytes. The cells were treated with or without TNFα and the luciferase activity was measured. Significant increases (>10 fold) in the expression from the *YKL40* promoter deletion constructs were observed in the cells treated with TNFα compared to untreated (Figure 2D). However, deletion of the −1000 to −500 bp region of the *YKL40* promoter impaired TNFα mediated transcriptional induction. This indicates that this region (−1000 to −500 bp) contains binding sites for transcriptional regulatory elements on the *YKL40* promoter.

### Computational Prediction of Transcription Factors Regulating *YKL40* Expression

In order to identify transcription factors that regulate the expression of *YKL40* a computational analysis of −4000 bp upstream of the open reading frame was done using the transcription element search system. The program predicted consensus binding sites in the DNA for several transcription factors that included NFKB subunit P65 and CEBPα (Figure 3A). The software predicted a consensus binding site for p65 (GGAATTTCC) at −578 bp position at the promoter. Similarly several DNA binding sites for CEBPα (CCAAT) was also predicted throughout the *YKL40* promoter. Most of the CEBPα binding sites were concentrated in the −3000 to −2000 bp region of the promoter. Interestingly, two CEBPα binding sites were identified in close proximity to the P65 DNA binding site on the promoter (Figure 3A) suggesting that these two transcription factors may bind to the *YKL40* promoter by forming a transcriptional regulatory complex.

### Mutation of NFKB (P65) Binding Site Inhibits TNFα Mediated *YKL40* Induction

Computational analysis predicted a putative binding site for NFKB subunit P65 at −578 bp position of the human *YKL40* promoter. Further the *YKL40* promoter analysis using the deletion constructs identified −1000 to −500 bp region to be essential for TNFα mediated induction of *YKL40* in hepatocytes. To test whether P65 binding plays a role in TNFα mediated upregulation of *YKL40* promoter a −578 bp wildtype luciferase reporter construct, a −578 bp construct with point mutations in the P65 binding site (wildtype: GGAATTTCC, point mutation: AGCTTATCA) and a −500 bp construct with P65 binding site deleted were prepared. Hepatocytes were transfected with these three reporter constructs and cells were treated either with or without TNFα and Luciferase activity was measured. As expected the wildtype −578 bp promoter construct with an intact P65 binding site showed two fold increase in transcriptional activity in the presence of TNFα (Figure 3B). However, point mutation in the P65 binding site or deletion of the P65 binding site (−500 bp) completely abolished TNFα mediated induction of *YKL40* (Figure 3B). Further, we demonstrated that siRNA mediated knockdown of P65 resulted in impairment of transcriptional activation by the *YKL40* promoter in TNFα treated cells.

### CEBPα in an Upstream Transcription Factor which Activate *YKL40* Expression

Computational analysis identified CEBPα as a putative DNA binding factor for transcriptional regulation of *YKL40*. To test whether CEBPα regulates *YKL40* expression an empty vector or a vector expressing CEBPα was overexpressed in hepatocytes along with deletion mutants of the *YKL40* promoter cloned upstream of a luciferase reporter gene. The deletion mutants included −3000 bp, −2000 bp, −1000 bp and −500 bp of the *YKL40* promoter region (Figure 3C, black filled bars on the left) and the luciferase activity was measured after the cells were treated with TNFα for 6 hours. Significant increases (>9 fold) in the *YKL40* expression from the −3000 bp promoter construct was noted when CEBPα was overexpressed compared to control empty vector (Figure 3C). However, deletion of the −3000 bp to −2000 bp region of the *YKL40* promoter impaired CEBPα mediated transcriptional induction of *YKL40* as that region contained several putative CEBPα binding sites as shown on our computational analysis (Figure 3A).

### CEBPα Interacts with NFKB/P65 to Bind *YKL40* Promoter and Cooperates to Activate Transcription in Hepatocytes

Reporter analysis demonstrated that both P65 and CEBPα regulate expression from the *YKL40* promoter. To test whether P65 and CEBPα interact and bind to adjacent consensus sites present on the *YKL40* promoter a chromatin immunoprecipitation analysis was done. TNFα treated hepatocytes were fixed with formaldehyde and chromatin extracts were prepared. The DNA-protein complexes were immunoprecipitated with anti-P65 or anti-CEBPα or isotype control IgGs. Chromatin fragments were isolated from the immunoprecipitated DNA-protein complexes and subjected to PCR amplification using primers specific for *YKL40* promoter regions as shown in Figure 4A. The PCR products were resolved on agarose gel. Chromatin immunoprecipitation analysis showed that both P65 and CEBPα bind to the *YKL40* promoter (Figure 4B). P65 binds to the −300 bp to −700 bp region of the *YKL40* whereas no binding was observed with other regions of the promoter. This region encompasses the P65 binding site at −578 bp position. CEPBα showed binding to several regions of the *YKL40* promoter where maximum band intensity was seen at −1900 bp to −2300 bp region that encompasses most of the CEBPα binding sites. Immunoprecipitation with isotype IgGs did not enrich any of these *YKL40* promoter regions demonstrating the specificity for P65 or CEBPα. No amplification of DNA was observed in PCR reactions performed with primers specific for *ACTIN* promoter.

Binding of both CEBPα and P65 to the *YKL40* promoter in adjacent DNA binding sites suggests the possibility that they interact with each other. To test this, crude lysates from TNFα treated hepatocytes were prepared and subjected to immunoprecipitation with either isotype control IgG or anti-P65 followed by immunoblotting with anti-CEBPα. CEBPα was co-immunoprecipitated with endogenous P65 whereas no CEBPα was observed with the control IgG (Figure 4C). To confirm the interaction a reverse co-immunoprecipitaion was done by immunoprecipitation with either isotype control IgG or anti-CEBPα followed by immunoblotting with anti-P65 (Figure S1). To further demonstrate the interaction between P65 and CEBPα, HEPG2 cells were treated with or without TNFα and co-immunostained with anti-CEBPα and anti-P65. CEBPα primarily localized to the nucleus in both TNFα treated and untreated cells. P65 remained exclusively cytoplasmic in the untreated cells with little to no nuclear localization. In the TNFα treated cells, a significant amount of P65 translocated into the nucleus and co-localized with CEBPα (Figure 4D).

Immunoprecipitation and co-localization analyses indicated that both P65 and CEBPα interact with each other and bind to their consensus sites on the *YKL40* promoter. To determine their role in transcriptional regulation from the *YKL40* promoter, hepatocytes were co-transfected with a luciferase reporter construct driven by the *YKL40* promoter (−3000 bp) with P65 or CEBPα or both. Overexpression of both P65 and CEBPα resulted in significant increase in transcriptional activation of the reporter construct compared to either alone (Figure 4E, upper panel). Immunoblotting of the cell lysates with anti-P65 and anti-CEBPα confirmed the elevated expression levels of these factors compared to endogenous levels (Figure 4E, lower panel). This demonstrates that CEBPα cooperates with NFKB to regulate expression from the *YKL40* promoter.

### NOTCH1 Regulates Nuclear Retention of NFKB/P65 in Response to TNFα

Results presented clearly demonstrates that TNFα mediated regulation of *YKL40* is dependent on the NFKB subunit P65. To demonstrate nuclear translocation of P65 in response to TNFα in hepatocytes, cells were treated with or without TNFα and cytoplasmic and nuclear extracts were prepared. Whole cell lysates, the cytoplasmic fraction and the nuclear fraction were subjected to immunoblotting with anti-P65. The expression of P65 was not affected by TNFα as no difference was observed in the whole cell lysate (Figure 5A, left panel). However, significant cytoplasmic exclusion and nuclear enrichment of P65 was observed in TNFα treated cells compared to untreated cells. One of the essential upstream regulators of NFKB complex is NOTCH1 [11], [12]. To analyze the role of NOTCH1, an upstream factor required for TNFα mediated nuclear localization of P65, and its functionality in facilitating downstream gene regulation, hepatocytes were transfected either with scrambled siRNA or siRNA specific for human *NOTCH1* and subjected to TNFα treatment. The siRNA mediated knockdown of *NOTCH1* was confirmed by immunoblotting with anti-Notch1 (Figure S2A). Immunoblot using anti-P65 demonstrated that knockdown of *NOTCH1* resulted in impairment of TNFα mediated cytoplasmic exclusion and nuclear translocation of P65 (Figure 5A, right panel).

To further demonstrate that NOTCH1 is required for TNFα mediated translocation of P65, HEPG2 cells were treated with or without TNFα and probed for P65 localization by immunostaining with anti-P65 (Figure 5B). In the untreated cells P65 remained exclusively cytoplasmic with little to no nuclear localization observed. In the TNFα treated cells, a significant amount of P65 translocated into the nucleus and quantification showed more than three fold nuclear abundance of P65 compared to the untreated cells (Figure 5C). Next, the HEPG2 cells were transfected with either scrambled siRNA or siRNA specific for human *NOTCH1* and subjected to TNFα treatment. Immunostaining with anti-Notch1 showed more than four fold knockdown of *NOTCH1* by siRNA (Figure S2B, C). Knockdown of *NOTCH1* resulted in impairment of TNFα mediated nuclear translocation of P65 by two fold (Figures 5D and E). TNFα mediated nuclear localization of P65 in cells transfected with scrambled siRNA was not affected. This finding indicates that NOTCH1 acts as an upstream regulatory factor and controls TNFα mediated nuclear translocation of P65.

### miRNA-449a Regulates *YKL40* Expression by Modulating *NOTCH1* Expression

Genomewide microarray analysis in our laboratory followed by miRNA gene expression analysis showed that miRNA-449a is downregulated more than two fold in HCV patients compared to non-HCV liver diseases and normals (Figure 1A). Computational target prediction of miRNA-449a using Targetscan (Targetscan.org) identified *NOTCH1* to be a putative target for translational silencing. To test this, hepatocytes were transfected with either empty vector of vector expressing miRNA-449a and expression of both miRNA-449a and NOTCH1 were determined by qPCR. The results were normalized to *GAPDH* expression. Increased expression of miRNA-449a resulted in more than two fold downregulation of *NOTCH1* (Figure 6A and B).

Earlier we have shown that NFKB component P65, a protein regulated by NOTCH1, activates *YKL40* expression through sequence specific promoter interaction (Figures 3 and 4). It is likely that downregulation of miRNA-449a in HCV infected patients (Figure 1A) results in activation of NOTCH1/NFKB signaling that leads to upregulation of *YKL40* expression. To test whether miRNA-449a regulates *YKL40* expression, hepatocytes were transfected with either a control vector or vector expressing miRNA-449a along with an *YKL40* promoter-driven luciferase reporter construct. In TNFα treated cells expression of *YKL40* is reduced by more than two fold in the presence of miRNA-449a (Figure 6C, upper panel). qPCR analysis also showed downregulation of *YKL40* by increased expression of miRNA-449a (Figure S3).

Since, computational target analysis did not identify *YKL40* to be a direct target for miRNA-449a; results obtained from promoter based reporter analysis suggest that miRNA-449a regulates the expression of *YKL40* by silencing components of upstream transcriptional regulatory complexes such as NOTCH1/NFKB. To demonstrate that downregulation of *YKL40* expression by miRNA-449a is mediated by silencing *NOTCH1,* hepatocytes were transfected with either scrambled siRNA or siRNA specific for *NOTCH1* along with an *YKL40*-driven luciferase reporter construct and cells were treated with TNFα. siRNA mediated knockdown of *NOTCH1* impaired expression from the *YKL40* promoter (Figure 6C, upper panel). We have demonstrated that nuclear P65, regulated by NOTCH1, activates *YKL40* expression in response to TNFα. siRNA mediated knockdown of P65 also impaired expression from the *YKL40* promoter (Figure 6C, upper panel). Western blot analysis using anti-YKL40 showed downregulation of *YKL40* by expression of miRNA-449a or siRNA mediated knockdown of *NOTCH1* or P65 in hepatocytes (Figure 6C, lower panels). qPCR analysis also confirmed downregulation of *YKL40* by miRNA-449a (Figure S3). Immunoblot analysis using anti-NOTCH1 demonstrated that expression of miRNA-449a resulted in downregulation of NOTCH1. Since, P65 is a downstream factor for NOTCH1, knockdown of P65 did not affect its expression (Figure 6C, lower panels). This suggests that miRNA-449a regulates expression of *YKL40* by modulating the NOTCH1 signaling pathway.

To further determine if TNFα mediated activation of *YKL40* is regulated by miRNA-449a, hepatocytes were transfected with either a control vector or vector expressing miRNA-449a along with an *YKL40*-driven luciferase reporter construct (−3000 bp) and cells were treated with or without TNFα. In the cells expressing the empty vector, TNFα induced expression from the *YKL40* promoter (Figure 6D). However, in presence of miRNA-449a this TNFα mediated upregulation of *YKL40* was impaired.

### *NOTCH1* Expression is Upregulated in HCV Patients

To determine whether downregulation of miRNA-449a in HCV infection is accompanied by upregulation of its target *NOTCH1*, biopsies were obtained from 10 chronic HCV patients, 10 alcoholic hepatitis patients, 10 NASH patients and 10 control normal donor livers at the time of liver transplant. Total RNA isolation followed by qPCR demonstrated *NOTCH1* to be significantly upregulated in livers obtained from HCV patients. However, no significant difference in the expression of *NOTCH1* was observed in alcoholic hepatitis patients, NASH patients and normal livers (Figure 7). Based on our *in-vitro* results obtained with hepatocytes, upregulation of *NOTCH1* and *YKL40* (Figure 1) in HCV patients can be attributed to downregulation of miRNA-449a.

## Discussion

YKL40, a member of the mammalian chitinase-like protein, has been shown to be elevated in patients with chronic liver diseases with fibrosis and cirrhosis (Figure 1) [5], [6]. In chronic liver disease patients, YKL40 expression has been shown to have a strong correlation with degree of fibrosis progression, extracellular matrix (ECM) synthesis, and serves an early indicator of liver fibrosis [23], [24]. In HCC patients, YKL40 expression is highly elevated in both serum and liver tissue [25]. Here we demonstrate that *YKL40* expression in human livers is regulated by co-operative action of several promoter-bound transcription factors. By computational analysis and subsequent *in vitro* studies we have defined putative binding sites for NFKB subunit P65 and CEBPα in the *YKL40* promoter (Figure 3A). The NFKB pathway plays an important role in liver fibrosis, as its activation in hepatocytes can lead to activation of surrounding tissue macrophages and thus leading to fibrosis [26]. We demonstrated that TNFα mediated *YKL40* expression is regulated by P65, a component of the NOTCH/NFKB signaling pathway (Figures 2 and 3). Additionally our study demonstrates that NOTCH1 is essential for nuclear retention of P65 in human liver cells (Figure 5). Studies in animal models have shown that knockdown of *NOTCH1* resulted in impairment of DNA binding and transcriptional activation ability of P65 and impacted dendritic cell differentiation [12]. We have shown for the first time that the NFKB subunit P65 cooperates with CEBPα to regulate expression of *YKL40* through direct DNA binding in hepatocytes (Figures 3 and 4). Several studies have shown that CEBPα regulates activation of hepatic stellate cells which play key roles in hepatic fibrosis [27], [28]. Differential modulation of CEBPα has been shown in HCC patients [17], [29]. Our analysis on the regulation of the inflammatory biomarker *YKL40* expression at the transcriptional level provides new insight into role of components of the NOTCH and NFKB signaling pathways in HCV induced hepatic fibrosis and HCC. It is of interest that HCV core protein NS3 can activate the NOTCH signaling pathway resulting in development of HCV-induced HCC [30]. Activation of NOTCH signaling also promotes TGFβ1 induced epithelial-mesenchymal transition, an initial step in the development of fibrosis, by directly interacting with the transcriptional machinery [31], [32], [33]. Modulation of both NOTCH1 and NFKB pathways have also been implicated in several cancers including HCC [34], [35], [36], [37].

In addition to demonstrating the interaction of NOTCH, CEBPα and NFKB pathways in *YKL40* expression, we determined an important role for modulation of miRNA by HCV. The understanding of the complex role of miRNAs in the various physiological and pathological processes is still emerging. miRNAs have been implicated in regulation of pro-inflammatory cytokines, anti-inflammatory cytokines, and interferons [38]. miRNA-21 has been shown to regulate chronic rejection and has been implicated in the development of fibrosis following liver transplantation [39], [40]. miRNA-21 modulates resident fibroblasts, epithelial cells and lymphocytes to produce pro-fibrotic cytokines resulting in deposition of ECM components [39], [41]. It has been shown that several liver specific miRNAs including miRNA-122, miRNA-148, miRNA-194 are sensitive biomarkers for hepatocyte injury and rejection after liver transplantation [42]. In this study we identified a novel miRNA (miRNA-449a) that is modulated in HCV infection. Further, we have shown that miRNA-449a regulates HCV induced inflammatory responses (YKL40) implicated in allograft liver fibrosis.

Previous studies in our laboratory have demonstrated an upregulation of autoimmune Th17 inflammatory cascade leading to liver fibrosis in HCV infection, particularly in recurrent HCV following orthotopic liver transplantation [43]. Viral modulations of miRNA have been well known to influence transcriptional regulation in T cell responses, inflammation and fibrosis [44]. Although suggested in literature, a direct effect of HCV mediated modulation of cellular inflammatory responses and fibrosis is yet to be determined. In our current study using promoter analyses techniques we provide direct evidence for the role of miRNA-449a, which is down regulated in HCV infection (Figure 1), in the upregulation pro-inflammatory YKL40 fibrotic cascade. miRNA-449a has been implicated transcriptional dysregulation affecting cell proliferation in several human diseases including cancers [45], [46]. *In vitro* studies have also shown that miRNA-449a can arrest cell proliferation and induce apoptosis [46], [47]. Thus, HCV induced down regulation of miRNA-449a in human livers can upregulate transcriptional factors leading to increased inflammatory response; promoting cell proliferation that can result in HCC.

We have also demonstrated by *in vitro* analysis that miRNA-449a regulates TNFα mediated induction of *YKL40* by targeting components of the NOTCH signaling pathway (*NOTCH1*) (Figure 6). We have shown for the first time in human hepatocytes that miRNA-449a targets *NOTCH1* for translational silencing. Studies have shown that miRNA-34a is downregulated in patients with chronic hypoxia kidney diseases and promotes epithelial-mesenchymal transition by targeting components of the NOTCH signaling pathway [48]. In HCV infected patients expression of miRNA-449a was significantly downregulated (Figure 1A). In consistence with our *in vitro* results, in the same HCV patients downregulation of miRNA-449a was accompanied by significant upregulation of *NOTCH1* (Figure 7). Thus, results obtained from patient samples and our *in vitro* analysis using hepatocytes indicate that upregulation of *NOTCH1* resulting from downregulation of miRNA-449a stabilizes nuclear P65 to activate *YKL40* expression in patients with HCV mediated hepatic fibrosis (Figure 1). The increased expression of *YKL40* in patients with HCV mediated liver fibrosis [5], [6] can be attributed to this novel pathway (Figure 8). Since, YKL40 is elevated in patients with multiple liver diseases (Figure 1B); it is likely that other parallel pathways for its transcriptional regulation may exist in non-HCV mediated liver fibrosis.

Taken together our results provide new insight into the mechanisms by which miRNAs mediate changes in the inflammatory process by modulating components of the transcriptional machinery. The results from this study should assist in the development of novel strategies for identifying non-invasive biomarkers that can prognosticate patients and monitor those at increased risk for development of cirrhosis and HCC following HCV infection.

## Supporting Information

Figure S1 **CEBPα interacts with P65.** Co-immunoprecipitation of CEBPα with P65 in hepatocytes. Whole cell lysates were subjected to immunoprecipitation with either mouse IgG or anti- CEBPα. P65 in the cell lysates (Input) and immunoprecipitated complexes (IP) was detected by immunoblotting with anti-P65. CEBPα was detected by immunoblotting with anti- CEBPα.
(TIF)

Figure S2 **siRNA mediated knockdown of** ***NOTCH1.*** A. Hepatocytes were transfected with either non-specific siRNA or siRNA specific for NOTCH1 and treated with TNFα. Lysates were subjected to immunoblotting with anti-NOTCH1. B. HEPG2 cells were transfected with either non-specific siRNA or siRNA specific for NOTCH1, treated with TNFα and immunostained with anti-NOTCH1. C. Quantification of the NOTCH1 immunostaining signal in HEPG2 cells (S2B). The numbers represent the average fluorescence intensity of P65 (n = 100).
(TIF)

Figure S3 **miRNA-449a regulates** ***YKL40*** **expression.** Hepatocytes were transfected with an empty vector (-) or vector expressing miRNA-449a (+) and expression of *YKL40* was determined by Q-PCR. The ΔΔ*CT* value was calculated by normalizing the threshold (CT) values with *GAPDH* and expression of *YKL40* in controls. The ‘*’ represents p value<0.05 obtained by a two-tailed t-test.
(TIF)
