# Supplementary figures and images for: Hepatitis C Virus Mediated Changes in miRNA-449a Modulates Inflammatory Biomarker YKL40 through Components of the NOTCH Signaling Pathway
Source: PLoS One. 2012 Nov 30;7(11):e50826. doi: 10.1371/journal.pone.0050826 (PMC3511274; doi:10.1371/journal.pone.0050826)

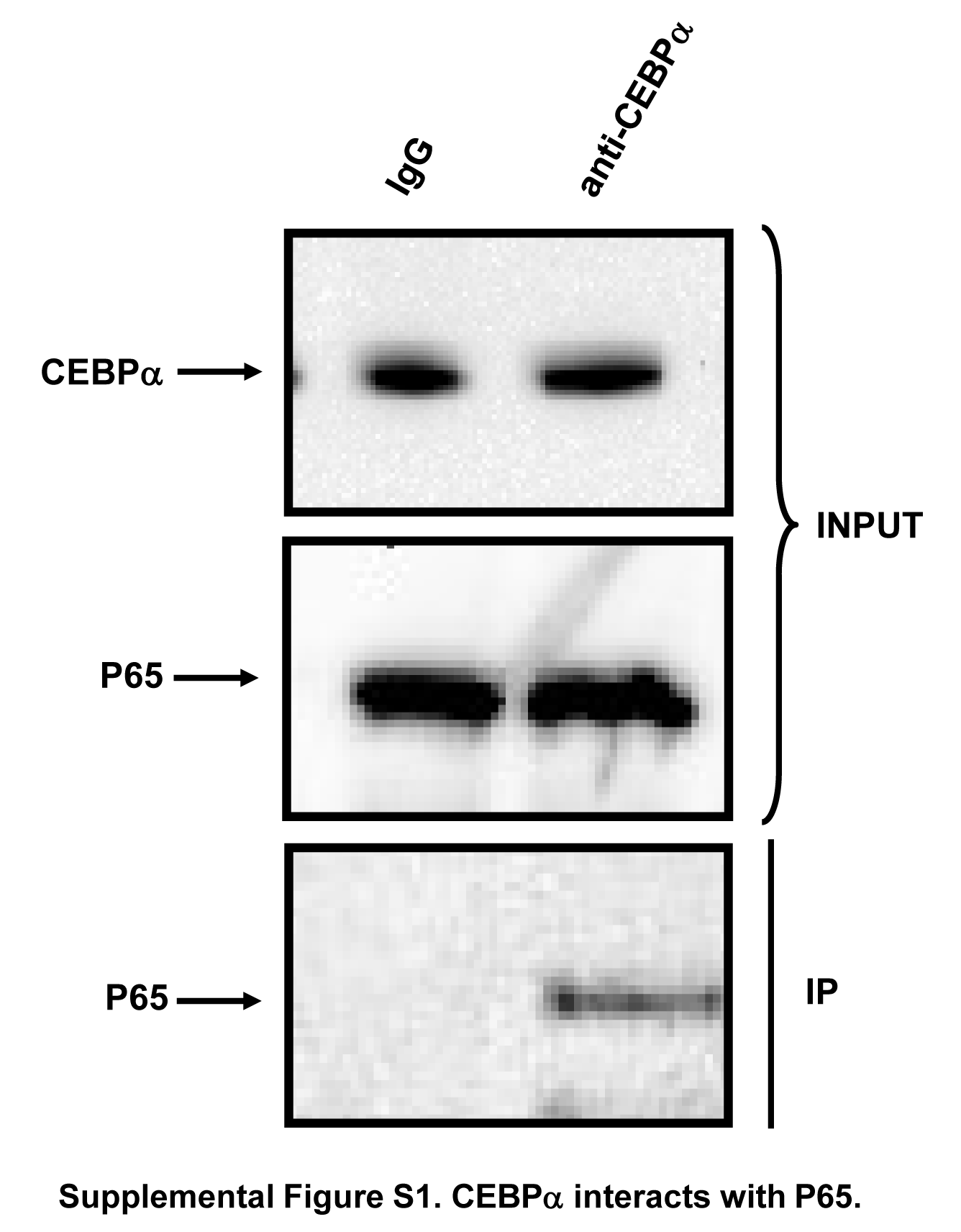

Supplement: Figure S1 — CEBPα interacts with P65. Co-immunoprecipitation of CEBPα with P65 in hepatocytes. Whole cell lysates were subjected to immunoprecipitation with either mouse IgG or anti- CEBPα. P65 in the cell lysates (Input) and immunoprecipitated complexes (IP) was detected by immunoblotting with anti-P65. CEBPα was detected by immunoblotting with anti- CEBPα. (TIF) [file pone.0050826.s001.tif]

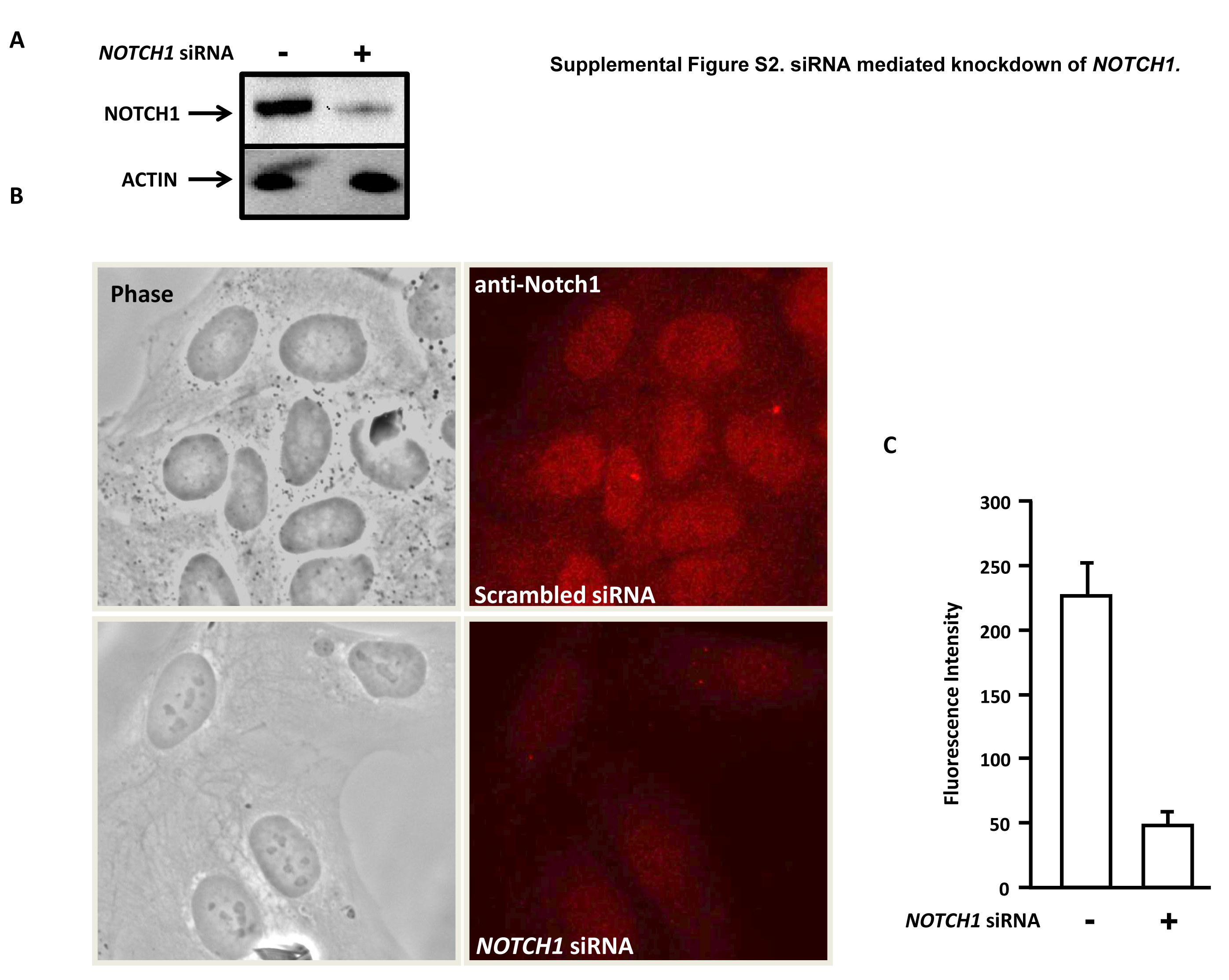

Supplement: Figure S2 — siRNA mediated knockdown of NOTCH1. A. Hepatocytes were transfected with either non-specific siRNA or siRNA specific for NOTCH1 and treated with TNFα. Lysates were subjected to immunoblotting with anti-NOTCH1. B. HEPG2 cells were transfected with either non-specific siRNA or siRNA specific for NOTCH1, treated with TNFα and immunostained with anti-NOTCH1. C. Quantification of the NOTCH1 immunostaining signal in HEPG2 cells (S2B). The numbers represent the average fluorescence intensity of P65 (n = 100). (TIF) [file pone.0050826.s002.tif]

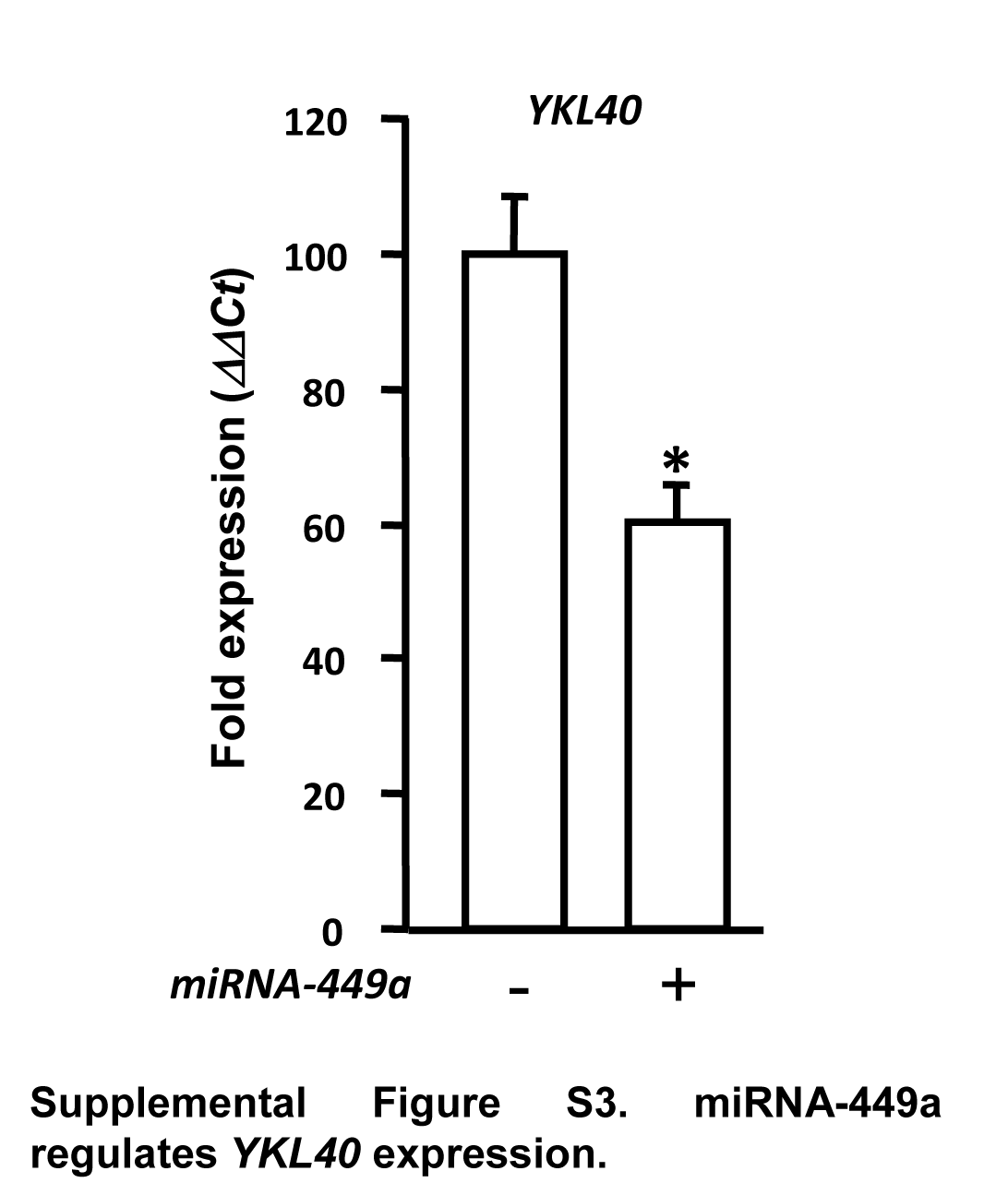

Supplement: Figure S3 — miRNA-449a regulates YKL40 expression. Hepatocytes were transfected with an empty vector (-) or vector expressing miRNA-449a (+) and expression of YKL40 was determined by Q-PCR. The ΔΔCT value was calculated by normalizing the threshold (CT) values with GAPDH and expression of YKL40 in controls. The ‘*’ represents p value<0.05 obtained by a two-tailed t-test. (TIF) [file pone.0050826.s003.tif]
